# The Influence of Ship Waves on Sediment Resuspension in the Large Shallow Lake Taihu, China

**DOI:** 10.3390/ijerph17197055

**Published:** 2020-09-27

**Authors:** Minsheng Bu, Yiping Li, Jin Wei, Chunyan Tang

**Affiliations:** Key Laboratory of Integrated Regulation and Resource Development on Shallow Lakes, Ministry of Education, College of Environment, Hohai University, Nanjing 210098, China; hjxyym@hhu.edu.cn (M.B.); weijin@hhu.edu.cn (J.W.); tangchunyan@hhu.edu.cn (C.T.)

**Keywords:** hydrodynamics, ship waves, sediment resuspension, wind-induced wave

## Abstract

Sediment resuspension induces endogenous nutrient release in shallow lakes, which has been demonstrated to be associated with eutrophication. In addition to natural wind-driven resuspension, navigable shallow lakes (such as Lake Taihu, China) also experience resuspension from human activities, such as ship waves. Both processes determine the intensity, frequency, and duration of sediment resuspension, and may consequently affect the pattern of cyanobacteria blooms in the lake. In this study, acoustic Doppler Velocimeter (ADV), Optical Backscatter Sensor (OBS), and temperature wave tide gauge (instrument model :RBR duo TD|wave) were placed in an observation platform in the lake to obtain high-frequency flow velocities, suspended sediment concentration (SSC), and wave parameters before, during, and after a cargo ship passed by. We found that the ship wave disturbance intensity is greatly influenced by the draft depth. The movement generated by ship disturbance is primarily horizontal rather than vertical. Compared with the wind-induced wave, the disturbance caused by the ship waves has a high intensity, short duration, and narrow range of influence. The maximum total shear stress under ship disturbance can reach 9~90 times the critical shear stress under a natural state. Therefore, the effect of ship waves on sediment resuspension near the channel of Lake Taihu is much greater than that of wind-induced waves. These findings represent an important step towards understanding the quantitative relationship between ship wave disturbance and sediment resuspension, and lay the foundation for future research in order to understand and control the eutrophication of shallow lakes.

## 1. Introduction

Eutrophication is regarded as one of the most urgent environmental problems in shallow lakes throughout the world [1]. A large number of studies have confirmed that revealing the driving mechanism of endogenous nutrient release under dynamic disturbance is the key to solving this problem [2]. Dynamic disturbance can be separated into natural disturbance caused by wind or high-speed water flow and human disturbance, including dredging, trawling, and navigation. At present, there are many examples of the study of sediment resuspension in shallow lakes which confirm that wind-driven wave is the most important power source of large shallow lakes [3]. For example, sediment suspension caused by wind-driven waves accounts for more than 70% of the total in Lake Taihu [4,5,6]. However, the research on ship waves mainly focuses on the observation of ship waves in inland waterway, inshore, or lakeside areas, or its influence on the stability of hull, wharf, and coastline [7,8,9], while the research on the mechanism of ship wave’s influence on the resuspension of shallow lake sediment and on the flotation, transport, and spatial accumulation of cyanobacteria is scant and has been overlooked. Therefore, this field will be the focus and hotspot in the field of the hydrodynamics and eutrophication of shallow lakes in the future.

The ship waves generated by large ships traveling in shallow water can cause sediment resuspension, and this is characterized by its regular occurrence, short duration, and high frequency [10,11]. The mass of resuspended sediment caused by ship wave disturbance can be higher than that caused by wind waves by more than one order of magnitude, which can increase the concentration of suspended solids in water by about 30 times [12]. However, the characteristics of sediment resuspension caused by wind-driven waves are accidental and random, with a large impact range and relatively long duration [13]. The combined action of ship waves and wind-driven waves will form irregular waves and currents, which will make the water disturbance more intense and the influence range, influence degree, and duration of sediment resuspension larger and more complex than those when ship waves or wind-driven waves act alone [14]. In navigable shallow lakes, ship waves and wind-driven wave often cooccur, and together determine the strength and duration of sediment resuspension and the degree of endogenous nutrient release.

**Figure 1:**
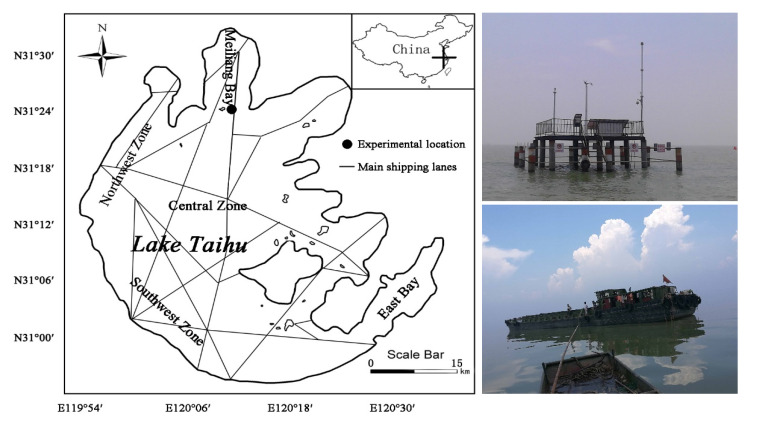
Experimental location, platform, and the chartered ship.

Therefore, how to determine the weight and relative contribution of ship waves and wind-driven waves to the spatial distribution of sediment resuspension and the potential effects on eutrophication in shallow water lakes is the basis and premise of guiding ecological channel construction and internal pollution control. Lake Taihu, the third largest freshwater lake in China, is well known as a large (2338 km^2^), shallow (mean depth = 1.9 m), and hyper-eutrophic lake. In the past few decades, Lake Taihu has been plagued by cyanobacteria due to excessive nutrient loading, which is exacerbated by endogenous release related to sediment resuspension [15,16,17]. Lake Taihu is also a busy navigable lake (Figure 1), serving the functions of waterway transportation, tourism, and fishery. According to the data of the Lake Taihu authority in 2018, The navigation capacity of the Taihu River Basin is about 200,000 ships per year. Lake Taihu is easily affected by both wind-induced waves and ship-induced waves, which is an ideal area for this study. In this research, relevant hydrodynamic parameters were obtained by simulating ships passing near the Taihu channel through experiments. The present study aims to explore the hydrodynamic characteristics and sediment behavior during the ship passages, and illuminate the mechanism of sediment resuspension caused by ship-induced waves and wind-induced waves.

## 2. Methods and Materials

### 2.1. Field Experiments

The field experiments were conducted on 28 July 2018 at the outlet of Meiliang Bay (31°22′56″ N, 120°9′38″ E) (Figure 1), which is the most seriously polluted area in Lake Taihu. The water depth of the observation point was 2.85 m. The lake bottom sediment consists of clay silt, with a median grain size of *d*_50_ = 12 µm. The three-dimensional, instantaneous flow velocities were measured using a SonTek Acoustic Doppler Velocimeter (ADV) with a sampling frequency of 100 Hz. Synchronous data of turbidity were obtained from a Campbell Optical Backscatter Sensor (OBS-3A) with a bursting interval of 1 min. Both ADV and OBS-3A were placed 5 cm above the lakebed. Water samples at the same depth were collected for a suspended sediment concentration (SSC) assay. Wave measurements were conducted using temperature wave tide gauges RBR duo TD|wave. The instrument was fixed 1 m below the water surface. It measured the significant wave height and wave period, the 1/10 wave height and wave period, the average wave height and wave period, the maximum wave height and wave period, and the wave energy with a sampling interval of 1 min. Wind data were acquired with the PH-II Handheld weather station.

**Figure 2:**
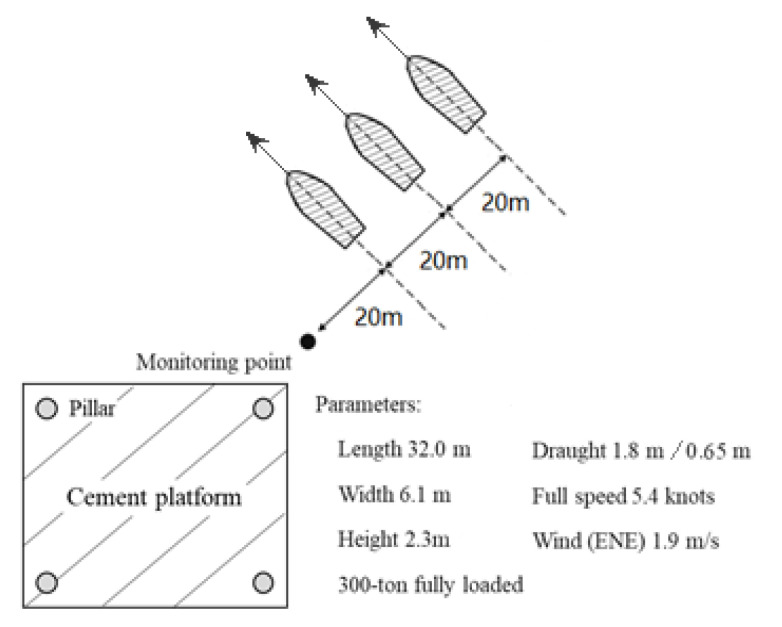
A sketch of the field experiments.

Through preliminary field research, we found that freighters are the most common ship in Lake Taihu. A 300-ton cargo ship with a length of 32.0 m, a width of 6.1 m, and a height of 2.3 m was hired for the field experiment (Figure 1). We tested several scenarios and focused on the most typical case—i.e., the ship passed 20, 40, and 60 m away from the monitoring point (Figure 2) at its maximum speed (i.e., 5.4 knots or 10 km/h) with a full load (draft 1.8 m) and no load (draft 0.65 m). The depth-based Froude number is *Fr_d_* = 0.53. To minimize the wind disturbance as much as possible, we chose a day with little wind (*v_wind_* = 1.9 m/s).

### 2.2. Data Analysis Methods

#### 2.2.1. High-Frequency Velocity Components Extraction

The ADV data were pre-processed by removing the outliers with low correlation coefficients (<70%) or low signal-to-noise ratios (<40 dB). To fill the missing data gaps (the time fraction of missing data was <1%), linear interpolation was used. Reynolds decomposition was used to extract the high-frequency (100 Hz) velocity components in the time series records [18].

#### 2.2.2. High-Frequency SSC Data Calculation

##### Laboratory Analysis of SSC

The turbidity (NTU) measured by the OBS was calibrated with the manually collected SSC (mg/L) data at the same time and same height. Every 3 h, 150 mL water samples were collected, filtered using 0.35 μm aperture membranes, and dried in the drying oven at 105 °C [19].

##### High-Frequency SSC

Echo intensity (EI) recorded by ADV was used as a representation of SSC according to the following equation [20,21]:

EI = A Log_10_ (SSC) + B. (1)

**Figure 3:**
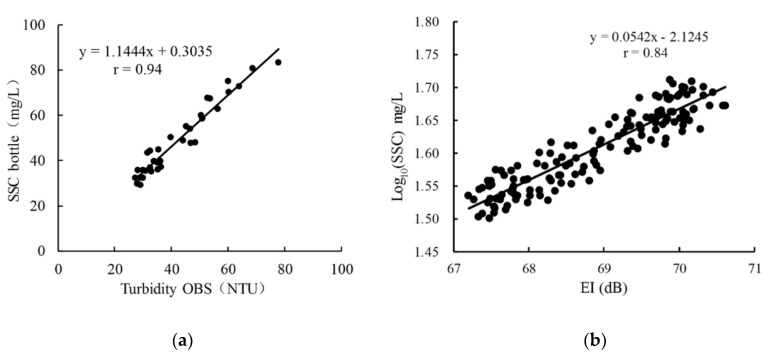
(**a**) Calibration between the turbidity (NTU) and SSC (mg/L) of bottle samples; (**b**) conversion of EI (dB) to high-frequency SSC (mg/L).

The collected water samples were used to convert the OBS turbidity (NTU) to SSC (mg/L), and the SSC data then were used to calibrate the EI. Figure 3 manifests strong relationships between the turbidity and SSC (r = 0.94), and EI and Log_10_ (SSC) (r = 0.84). High-frequency SSC data were calculated.

#### 2.2.3. Wave Data Processing Method

The wavelength is calculated according to the observed wave parameters:

(2) $$L_{s}=(\frac{{gT_{s}}^{2}}{2\pi })\tanh(\frac{2\pi h}{L_{s}}),$$

where *L_s_* is the significant wavelength, *T_s_* is the significant wave period, and *h* is the depth of the observation points.

The maximum orbital velocity of waves near the bottom layer *u_w_* (m/s) can be expressed as [22,23]:

(3) $$u_{w}=\frac{\pi H_{s}}{T_{s}\sinh\left(\frac{2\pi h}{L_{s}}\right)},$$

where *H_s_* is the significant wave height (m), *L_s_* is the significant wavelength (m), *T_s_* is the significant wave period, and *h* is the depth of observation points (m).

#### 2.2.4. Shear Stress in the Near-Bottom Layer

The bottom shear stress generated by the wave–current interactions is the main dynamic force for sediment resuspension in Lake Taihu [14]. To separate the different contributions of waves and currents, the shear stress generated by the waves and currents at the water–sediment interface was calculated based on the linear wave theory and Karman–Prandtl logarithmic velocity distribution law.

The shear stress caused by waves at the water–sediment interface can be calculated by the following equation [24]:

(4) $$\tau_{w}=0.5\rho f_{w}u_{w}^{2},$$

where *τ_w_* is the wave shear stress (N/m^2^), *ρ* is the water density (kg/m^3^), *u_w_* is the maximum wave orbital velocity near the bed (calculated by Equation (2)), and *f_w_* is the wave friction coefficient related to the lake bottom roughness and Reynolds number. The *f_w_* is calculated as follows [25]:

(5) $$f_{w}=\exp\left[5.2{\left(A_{\delta }/K_{s}\right)}^{-0.19}-6.0\right],\;f_{w,\max}=0.3\;\mathrm{if}\;A_{\delta }/K_{s}\leq 1.57,$$

where *K_s_* is the physical roughness of the lake bottom, which it is difficult to get a true value for using field observation. This study used 0.2 mm as the lake bottom roughness based on previous studies [14,19,26,27] and *A_δ_* is the amplitude of wave-particles (m), which is determined by the linear wave theory. *A_δ_* is calculated as follows:

(6) $$A_{\delta }=\frac{H_{s}}{2\sinh\left(\frac{2\pi }{L_{s}}h\right)}.$$

The shear stress caused by currents at the water–sediment interface is calculated as follows [26,28]:

(7) $$\tau_{c}=\rho u_{b}^{*}u_{b}^{*},$$

(8) $$u_{b}^{*}=\frac{ku_{z}}{\ln\frac{z}{z_{0}}},$$

where *τ_c_* is the shear stress caused by currents (N/m^2^); *u_b_* * is the friction velocity (m/s); *k* is the Kaman constant (0.4); *u_z_* represents the velocity, which is the height, *z*, above the bottom; and *z*_0_ is the bottom physical roughness (0.2 mm) [14,26,27]. The measurements by ADV are averaged over an integer number of wave periods in order to gain the sole contribution of the current to the velocity.

The maximum total shear stress induced by wave–current interactions is found as follows [29]:

*τ* = *τ_c_* + *τ_w_*, (9)

(10) $$\tau =\tau_{w}\sqrt{\left[1+{\left(\tau_{c}/\tau_{w}\right)}^{2}+2(\tau_{c}/\tau_{w})|\cos(\delta -\alpha )|\right]},$$

where *α* and *δ* are the directions of the waves and current, respectively.

## 3. Results

### 3.1. Hydrodynamic Characteristics and Sediment Behaviors Caused by Ship Waves

**Figure 4:**
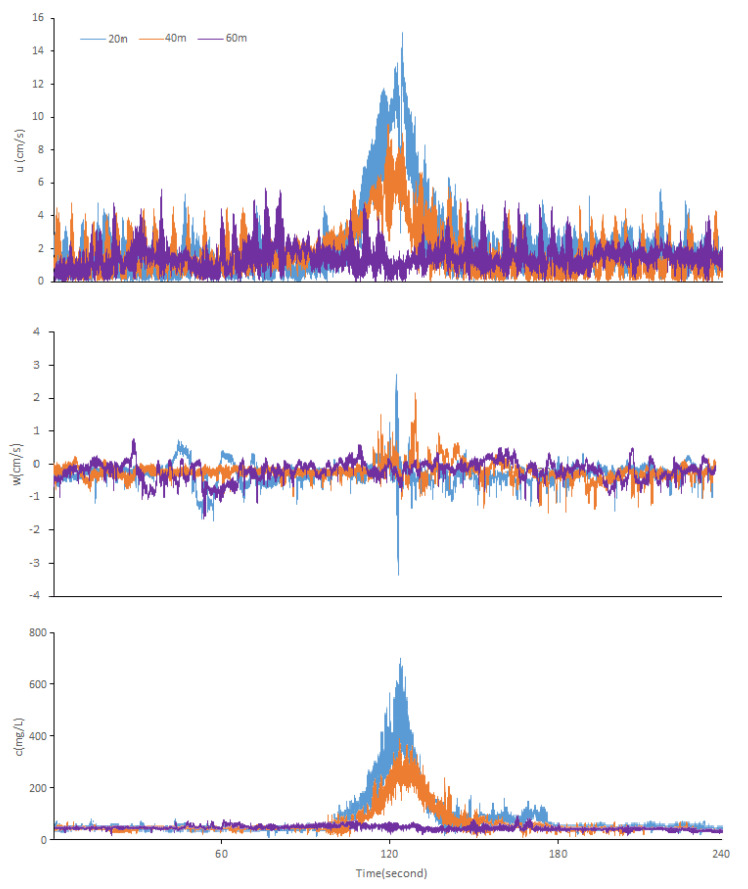
Time series records (full load): horizontal velocity (*u*), vertical velocity (*w*), and SSC (*c*) during the ship’s crossing.

**Figure 5:**
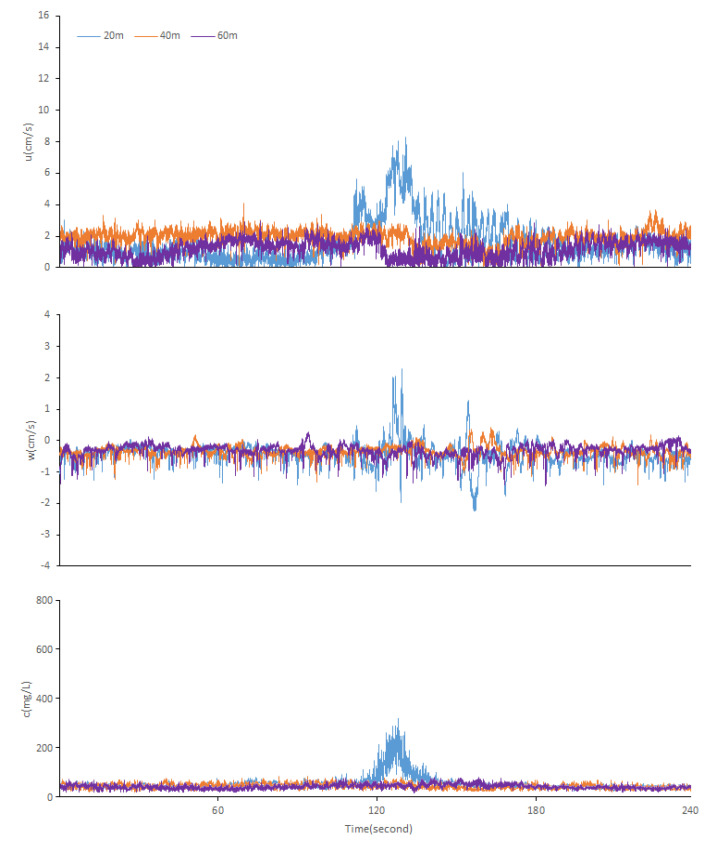
Time series records (no load): horizontal velocity (*u*), vertical velocity (*w*), and SSC (*c*) during the ship’s crossing.

Two minutes of data were taken before and after each passage for comparison. The changes in each parameter value in the process of the ship passing under the two states of full load and no load are shown in Figure 4 and Figure 5, respectively. Before the ship’s crossing under a full load, the horizontal flow velocity (*u*) varied from 0.01 to 4 cm/s. When the ship passed by, the horizontal flow velocity (*u*) reached up to 15.14 and 9.59 cm/s when the distance was 20 and 40 m, respectively (Figure 4). When the distance is 60 m under a full load, the horizontal velocity (*u*) of the ship before and after passing does not change significantly, indicating that the observation point is of the influence range of the ship waves at this time.

Before the ship’s crossing by under no load, the horizontal flow velocity (*u*) varied from 0.01 to 2.25 cm/s. When the ship passed, the horizontal flow velocity (*u*) reached up to 8.24 cm/s when the distance was 20 m (Figure 5). Meanwhile, the horizontal velocity (*u*) of the ship does not change significantly before and after the passing, when the distance is 40 and 60 m under no load. According to previous studies in Lake Taihu [30], the critical velocity of the sediment resuspension is 3–4 cm/s, which confirmed that the disturbance of ship waves hardly causes sediment resuspension at this time.

The vertical flow velocity (*w*) experienced a short-term rapid change between −3.56 and 2.59 cm/s during the crossing when the distance is 20 m under a full load, whereas it changed very little (−1.29~0.13 cm/s) before the ship came. The vertical flow velocity (*w*) experienced a short-term rapid change between −0.17 and 2.18 cm/s during the crossing when the distance is 40 m under a full load, whereas it changed very little (−0.65~0.21 cm/s) before the ship came. When the distance is 60 m under a full load, the vertical flow velocity (*w*) has no significant change before and after the ship’s passage, which also confirms that the distance of 60 m is beyond the range of the ship waves.

The vertical flow velocity (*w*) experienced a short-term rapid change between −1.97 and 2.23 cm/s during the crossing when the distance is 20 m under no load, whereas it changed very little (−1.22~0.45 cm/s) before the ship came. When the distance is 40 and 60 m under no load, the vertical flow velocity (*w*) has no significant change before and after the ship’s passage, fluctuating very little between −1.17 and 0.18 cm/s all the time, which also proves that the effect of ship waves disturbance is negligible.

When the distance is 20 or 40 m under a full load, in response to the flow velocity, the SSC kept increasing and reached as high as 709 or 389 mg/L after remaining at a low value and barely fluctuating for a long period of time. When the distance is 60 m under a full load, the SSC has no significant change. When the distance is 20 m under no load, in response to flow velocity the SSC kept increasing and reached as high as 321 mg/L after remaining at a low value (about 49 mg/L) and barely fluctuating for a long period of time. When the distance is 40 and 60 m under no load, in response to flow velocity, the SSC has no significant change; this phenomenon further confirms the previous conclusion that the disturbance of ship waves hardly causes sediment resuspension at this time.

By comparing the data in the range of the ship waves, the most striking results are the difference in the duration of the ship’s influence on the horizontal (*u*) and vertical flow velocities (*w*). For example, when the distance is 20 m under a full load, the ship passage affected the horizontal flow velocity (*u*) for about 1 min, while the vertical flow velocity (*w*) only fluctuated significantly for less than 10 s, which indicates that ship waves contribute more energy in the horizontal direction than in the vertical direction. The sediment was also suspended in large quantities, lasting approximately 30 s before it settled.

### 3.2. Mechanism of Ship Wave-Induced Sediment Resuspension

**Figure 6:**
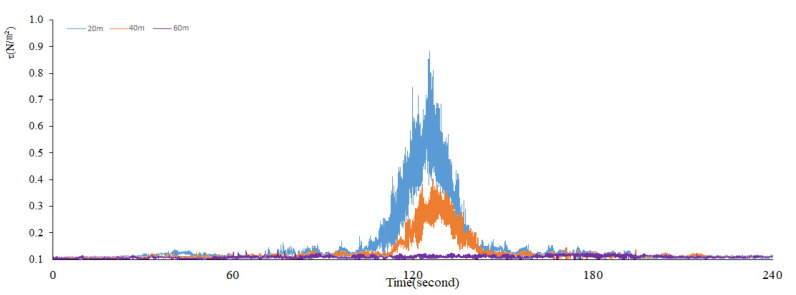
Changing trends of the maximum total shear stress (*τ*) under a full load.

**Figure 7:**
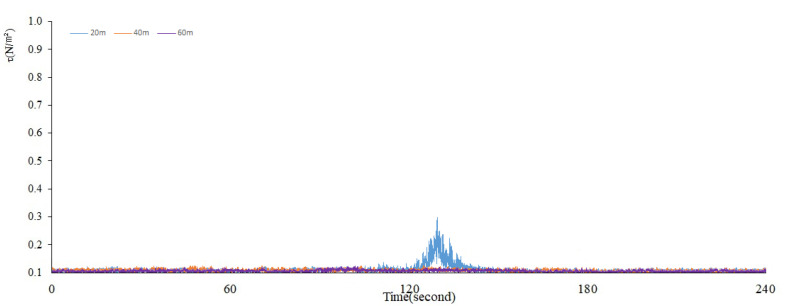
Changing trends of the maximum total shear stress (*τ*) under no load.

The maximum total shear stress caused by the disturbance was calculated based on Equations (4), (7), and (10).We can see the significant changes in the maximum total shear stress (*τ*) when the ship passed by under the full load (Figure 6) and no load (Figure 7).

When the distance is 20 m under a full load, the shear stress presents a more obvious normal distribution with time, and fluctuation lasts for about 30 s (Figure 6). The overall trend is relatively consistent with the SSC in Figure 4. When the ship passes through the observation point, the shear stress changes sharply, and the peak value can reach about 0.9 N/m^2^; previous studies have shown that the critical shear stress (the shear stress corresponding to the suspension of 5–10 cm sediment near the surface) range of sediment resuspension in Lake Taihu is about 0.01~0.1 N/m^2^ [14,31], which is roughly equivalent to the wind speed of 5~6 m/s. Therefore, the shear stress caused by the ship waves is about 9~90 times the critical shear stress in the natural state. At this time, the shear stress produced by ship waves plays a dominant role in the suspension of sediment. The shear stress also presents a more obvious normal distribution with time when the distance is 40 m under a full load. The fluctuation lasts for about 30 s, and the overall trend is relatively consistent with the SSC in Figure 4. The shear stress changes and the peak value can reach about 0.4 N/m^2^ while the ship passes through the observation point. The shear stress caused by the ship waves is about 4~40 times of the critical shear stress under the natural state at this moment. It can be seen from the figure that there is no obvious change in the shear stress. When the distance is 60 m under a full load, this is consistent with the change characteristics of SSC at this distance, again confirming that this distance has exceeded the influence range of ship waves.

Meanwhile, when the distance is 20 m under no load, the shear stress presents a normal distribution with time (Figure 7). The fluctuation lasts for about 30 s, and the overall change trend is relatively consistent with the SSC in Figure 5. When the ship passes through the observation point, the shear stress changes obviously, and the peak value can reach about 0.3 N/m^2^. At this time, the shear stress caused by the ship waves is about 3~30 times the critical shear stress under the natural state, and it has an important contribution to the suspension of sediment. When the distance is 40 and 60 m under no load, it can be seen from the figure that there is no obvious change in the shear stress at this time, which is consistent with the change in SSC at this distance, again confirming that the disturbance of ship waves little causes sediment resuspension at this time.

**Figure 8:**
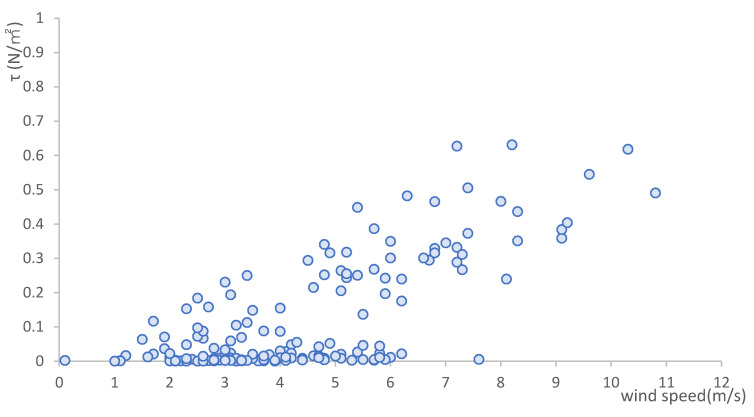
The relationship between the shear stress induced by wind and wind speed under natural conditions.

The previous research results of our research team under the natural disturbance of Lake Taihu [32] in Figure 8 shows the relationship between the wind-induced shear stress and wind speed under natural conditions. During the observation period, the wind speed in Lake Taihu area in the range of 2~6 m/s, the maximum wind speed is 10.8 m/s, and the maximum shear stress measured during the observation period is 0.63 N/m^2^. Compared with Figure 6 and Figure 7, the shear stress caused by the maximum wind speed is far less than that caused by the full load ship waves, which was equivalent to that caused by the no-load ship waves. According to our previous study [33], there was a strongly positive correlation between shear stress and nutrient release from sediment in Lake Taihu, and the formula of shear stress (*τ*) and nutrient release rate (*R*) near the bottom of Lake Taihu can be expressed as follows.

$$R_{TN}=0.1087e^{5.498\tau }\;(R^{2}=0.6433),\;R_{TP}=0.0221e^{4.9074\tau }\;(R^{2}=0.6132),$$

where *R_TN_* and *R_TP_* are *TN* and *TP* release rates, respectively. *τ* is the bottom shear stress.

There results suggest that strong shear stresses would be induced by the loaded ship waves, which subsequently could lead to intensity of sediment resuspension. With sediment resuspension, the increased nutrients in the overlying water and the algal cells from sediment can cause eutrophication [34,35].

## 4. Discussion

In our research, the influence of ship traveling wave within the channel is calculated. We found that the ship waves disturbance intensity is greatly influenced by the draft depth. Ship disturbance contributes most of the energy to the horizontal rather than the vertical direction. Compared with wind induced waves, the disturbance caused by ship waves has high intensity, short duration and narrow range of influence. The maximum total shear stress under ship disturbance can reach 9~90 times the critical shear stress under the natural state. Due to the large bottom shear stress caused by ship waves, nutrient release would increase near the channel of Lake Taihu, which may serve as an important induction factor to stimulate algal growth. Therefore, the release of nutrients caused by ship wave disturbance near the channel of Lake Taihu may be much greater than the wind-induced wave during the voyage of the ship. Thus, more attention should be paid to ship waves disturbance.

According to the data of Lake Taihu authority in 2018, the navigation volume in Lake Taihu Basin is about 200,000 ships per year. The peak season is from April to September every year, and the average monthly navigation volume in the peak season is about 23,000 ships. The peak season accounts for 70% of the total annual navigation volume, which coincides with the time of annual cyanobacteria proliferation in Lake Taihu. Based on the results obtained in the present study, the ship traveling wave induced by this huge navigation volume could exhibit strong impacts on the nutrients release and water environment of Lake Taihu, and may consequently affect the growth of cyanobacteria in the lake. These findings highlight the relationship between ship wave disturbance and sediment resuspension, and also have an important guiding value for controlling the release of endogenous nutrients and the eutrophication in Lake Taihu.

At present, the accelerated eutrophication of lake ecosystems is one of the major environmental concerns throughout the world. The sediment resuspension can regenerate nutrients and algae in sediment to overlaying water and accelerate the eutrophication [34,36]. A large number of studies have confirmed that dynamic disturbances, including wind, dredging, trawling, and navigation, can induce sediment resuspension with endogenous nutrient release in lake ecosystems. As a result, a sediment resuspension event, a lake internal process, will exacerbate the in return [35]. On the whole, they reveal the relationship between ship wave disturbance and sediment resuspension, and have important guiding value for the treatment of eutrophic lakes. The effects of sediment resuspension induced by ship waves should be also paid more attention in other shallow lakes with a high navigation volume, such as Chaohu Lake and Dianchi Lake.

## 5. Conclusions

The purpose of this study is to investigate the effect of ship waves caused by navigation on the sediment resuspension in large shallow lakes. Several conclusions can be drawn from this study:

(1) The ship wave disturbance intensity is greatly influenced by the draft depth, and ship disturbance contributes most of the energy to the horizontal direction rather than the vertical direction, which is manifested by both a longer duration of influence and a stronger turbulence intensity in the horizontal direction.
(2) Compared with the wind-induced wave, the disturbance caused by the ship waves has the characteristics of high intensity, short duration, and narrow influence range.
(3) The maximum total shear stress under ship disturbance can reach 9~90 times the critical shear stress under natural state. At this time, ship waves are the main factor causing sediment resuspension.
(4) Combined with our previous work, the cumulative impact of ship waves on sediment resuspension near the channel of Lake Taihu is much greater than that of wind-induced wave, and may affect the nutrient release and eutrophication in Lake Taihu.
